# Performance and safety of a fine-tuned small language model for pediatric emergency triage: A benchmark study

**DOI:** 10.1371/journal.pone.0350770

**Published:** 2026-06-04

**Authors:** Eui Jun Lee, Jae Yun Jung, Do Kyun Kim, Joong Wan Park, Young Ho Kwak

**Affiliations:** 1 Department of Emergency Medicine, Seoul National University College of Medicine, Seoul, Korea; 2 Department of Emergency Medicine, Seoul National University Hospital, Seoul, Korea; 3 Research Center for Disaster Medicine, Seoul National University Medical Research Center, Seoul, Korea; University of Hong Kong, HONG KONG

## Abstract

Pediatric emergency triage is a safety-critical task, and recent studies have explored whether artificial intelligence, including language models, can support triage decision-making; however, evidence on fine-tuned open-weight language models remains limited. We conducted a retrospective benchmark study using de-identified triage records from a tertiary pediatric emergency department in Korea collected from January 2020 to April 2025. After exclusions, 74,170 encounters were included. Each encounter was reconstructed into a case-level text sequence from triage-time structured variables and nurse-authored narratives. Qwen3-8B-Base was fine-tuned with Low-Rank Adaptation and Group Relative Policy Optimization using a safety-oriented reward design and was compared with a structured-data XGBoost model on a common evaluable test subset of 14,832 encounters. The fine-tuned model achieved an accuracy of 58.60%, a macro-F1 score of 0.417, and a quadratic weighted kappa of 0.535. Within-one-level agreement was 97.13%, and strict under-triage, defined as true Korean Triage and Acuity Scale levels 1 or 2 predicted as levels 4 or 5, occurred in 0.65% of cases. The structured-data comparator showed higher overall performance, with an accuracy of 69.40%, a macro-F1 score of 0.618, and a quadratic weighted kappa of 0.651. However, the fine-tuned model showed fewer extreme errors and lower strict under-triage in selected high-acuity groups, at the cost of higher over-triage. In this real-world pediatric benchmark, the fine-tuned language model did not surpass the structured-data comparator in overall performance but showed a distinct safety-oriented error profile. These findings support its potential role as a decision-support aid for human triage review rather than an autonomous triage system. External and prospective validation will be necessary before clinical implementation.

## Introduction

Triage serves as the initial phase of emergency care and plays a pivotal role in resource allocation, patient safety, mitigation of overcrowding, and timely treatment [1,2]. However, its effective implementation can be particularly challenging in specific clinical settings or patient populations. In pediatrics, communication is often limited, and assessments therefore rely largely on caregivers’ reports and clinicians’ observations [3,4]. Consequently, even experienced providers may have difficulty maintaining consistency, making pediatric triage inherently complex [5].

These challenges have prompted growing interest in artificial intelligence (AI) to support consistent and accurate triage decisions [6–10]. Prior work has included both structured-data models and language-based approaches for emergency triage, and recent multi-architecture benchmarks have shown promising performance in adult emergency department settings [11–13]. Large language models (LLMs) are of particular interest because they can process complex unstructured data, including caregiver reports and clinical notes, through chain-of-thought reasoning [14]. In emergency triage, LLM-based tools have shown potential to improve classification accuracy and reduce clinician cognitive load [6]. Their use has also been explored in pediatrics, where they may help identify injury patterns from emergency records [15].

Despite these advances, most studies have focused on very large models such as GPT-3 and GPT-4 [16]. These models face several barriers to clinical application, including substantial computational cost, privacy concerns, restricted domain adaptation, reduced reliability, and potential data contamination risks [17,18].

To address these limitations, smaller-scale language models have been proposed as lighter alternatives that may preserve multilingual support and reasoning capability [19,20]. Recent technical reports suggest that model capability is increasingly shaped not only by parameter count but also by distillation, data curation, and post-training, with smaller open-weight models showing increasingly competitive performance relative to much larger systems [21,22]. To further enhance their performance, optimization strategies such as retrieval-augmented generation and fine-tuning have been actively applied in biomedical contexts [23–28]. However, the real-world performance and safety profile of fine-tuned language models for emergency triage remain insufficiently characterized, particularly in pediatrics.

The objective of this study was to benchmark a fine-tuned language model for pediatric emergency triage against a structured-data comparator using real-world triage records. This assessment aimed to establish a realistic baseline for both the potential and risks associated with deploying such models in clinical decision-support roles.

## Methods

### Study design and settings

This retrospective study analyzed de-identified triage records collected in the pediatric emergency department of a tertiary hospital in Korea. We screened all visits recorded in this setting between January 2020 and April 2025. Encounters were excluded if triage acuity documentation was missing or if essential fields contained evident data-entry errors. The pediatric emergency department primarily serves patients aged 0–18 years, although a small number of older patients receiving ongoing pediatric care were also included.

### Data acquisition, cohort construction, and input definition

We accessed the hospital’s research query database to retrieve de-identified emergency department triage records that met prespecified eligibility criteria. The dataset was partitioned into training, validation, and test sets before model development. The test set was withheld until final evaluation, and a prespecified validation subset within the development pool was used during model development. For the primary analysis, the observed real-world triage class distribution was preserved without resampling; separate class-rebalanced experiments were conducted later as post-hoc methodological analyses.

Model inputs were limited to information available at the time of initial emergency department triage and were collected during routine care by triage nurses who had completed standardized triage training and competency assessment. The reference label was the nurse-assigned Korean Triage and Acuity Scale (KTAS) level, a five-level system ranging from 1 (most urgent) to 5 (least urgent), adapted from the Canadian Triage and Acuity Scale (CTAS) and implemented nationwide [29,30].

Each encounter comprised both structured fields and a free-text triage narrative. Structured variables included demographics, vital signs, arrival-related variables, past medical and surgical history fields, and the recorded chief complaint. The unstructured component was a nurse-authored narrative summarizing information obtained from the patient and caregiver at triage.

### Triage narrative reconstruction for language-model input

For language-model training and inference, each triage encounter was reconstructed into a single case-level text sequence using only information available at initial triage. Available structured fields and the nurse-authored triage narrative were combined into one case representation. The source text was retained in its original form to preserve routine clinical documentation, without manual correction, abbreviation expansion, translation, or normalization; missing fields were not added, inferred, or imputed. The triage notes were predominantly written in Korean and frequently contained English medical terms and abbreviations.

### Model architecture and primary training procedure

We fine-tuned Qwen3-8B-Base [31], an open-weight multilingual 8-billion-parameter language model. To enable parameter-efficient adaptation in a single-GPU setting, the base model was loaded in 4-bit precision and fine-tuned using Low-Rank Adaptation (LoRA) [24,32,33]. LoRA adapters were applied to the attention and feed-forward projection modules with rank r = 32, α = 64, and dropout = 0, yielding approximately 83 million trainable parameters. Key model and training hyperparameters, including the LoRA target modules, trainable parameter count, and wall-clock training duration, are provided in S1 Table in S1 File.

Optimization was performed using Group Relative Policy Optimization (GRPO), which updates the policy based on relative advantages among multiple candidate completions generated for the same prompt [34]. During training, four candidate completions were generated per prompt, and the primary configuration used a learning rate of 5 × 10^−6^, an effective batch size of 16, and a fixed random seed. After configuration selection during development, the one-epoch model was retained as the primary model based on development-stage training dynamics; a separate two-epoch class-rebalanced run was analyzed post hoc and is reported separately.

### Class imbalance handling and post-hoc training analyses

Because KTAS classes were markedly imbalanced, the primary training strategy preserved the empirical class distribution and did not apply resampling. The one-epoch model was retained as the primary benchmark model.

To examine whether class rebalancing or longer training materially changed model behavior, we conducted a separate post-hoc GRPO experiment using a rebalanced training set and extending training to two epochs. Because this secondary run altered both the training distribution and the training duration, it was treated as an exploratory methodological analysis rather than as a direct epoch-only comparison or a replacement for the primary model.

### Reward design and safety shaping

The reward function for five-level KTAS classification was designed to incorporate both prediction accuracy and clinical safety. Exact agreement with the reference KTAS level received the highest reward, whereas outputs that deviated from the reference received less favorable scores. Error penalties were asymmetric: under-triage was penalized more strongly than over-triage to reflect its greater clinical risk. An additional penalty was applied when true high-acuity cases (KTAS 1–2) were assigned low-acuity predictions (KTAS 4–5). Over-triage was penalized only modestly to avoid driving the model toward uniformly high-acuity predictions. The key reward components and coefficients used in training are provided in S2 Table in S1 File.

### Structured-data comparator: XGBoost

To provide a benchmark against a purely structured-data approach, we trained an XGBoost gradient-boosting classifier [35] using only structured variables available at the time of initial triage. Minority classes were weighted more heavily during training, and hyperparameters were selected using a held-out validation subset with early stopping. For fair comparison, the XGBoost model and the fine-tuned model were evaluated on the same common evaluable subset.

### Evaluation metrics and uncertainty estimation

The primary comparative analysis compared the pre-fine-tuning model, the final fine-tuned model, the structured-data XGBoost comparator, and a majority-class baseline on the same held-out test set, allowing direct assessment of performance change after fine-tuning. Language-model outputs were converted to KTAS labels using a deterministic parser that accepted only valid integer labels from 1 to 5. Responses that could not be resolved to a valid KTAS label were marked as parse failures. No fallback label, majority-class assignment, random assignment, or refusal-to-class conversion was applied. Parse failures were excluded from prediction-based metric calculations and reported separately as the parse failure rate.

Primary global performance metrics were overall accuracy, macro-averaged F1 score, and quadratic weighted kappa (QWK). The primary safety metric was strict under-triage, defined as true KTAS 1–2 cases predicted as KTAS 4–5. We report this both overall and separately for true KTAS 1 and true KTAS 2 cases. We also quantified misclassification across the emergent/non-emergent boundary, including true KTAS 1–3 cases predicted as KTAS 4–5 and true KTAS 4–5 cases predicted as KTAS 1–3. Secondary metrics included overall under-triage and over-triage rates, within- ± 1-level agreement, extreme error rate (absolute difference ≥2 levels), and class-level precision, recall, and F1 for each KTAS level. We also evaluated a majority-class baseline that assigned KTAS 3 to all encounters.

Percentile-based 95% confidence intervals were estimated by nonparametric bootstrapping with 500 resamples for the primary global metrics, safety-relevant error rates, and exploratory subgroup analyses. Model inference on the held-out test set was performed with temperature = 0. Exploratory subgroup analyses were stratified by age group and presenting symptom category; because subgroup sizes were unequal, these analyses were interpreted as exploratory and were not used for formal between-subgroup hypothesis testing.

### Compute environment and reproducibility

All experiments were conducted on a local Linux workstation (Ubuntu 24.04.3 LTS; Python 3.13) equipped with an AMD Ryzen 9 9950X CPU, 86 GB system RAM, and a single NVIDIA RTX 5090 GPU (34 GB VRAM).

### Ethics and data access

The study was conducted in accordance with the Declaration of Helsinki and was approved by the Institutional Review Board of Seoul National University Hospital (IRB No. E-2508-068-1665; approval date 14 August 2025). The requirement for individual informed consent was waived because this was a retrospective study using de-identified pediatric emergency department data. The data cannot be shared publicly because they contain potentially sensitive pediatric clinical information and are subject to institutional and ethical restrictions. Qualified researchers may request access through the Institutional Review Board Office of Seoul National University Hospital at irb@snuh.org, subject to review and approval in accordance with institutional policies. The source code used for model training and evaluation is publicly available at https://github.com/eklesia-lee/KTAS_GRPO. The study team accessed the data for research purposes on 15 August 2025, and the analytic dataset contained no direct identifiers.

## Results

### Study dataset and class distribution

**Fig 1:**
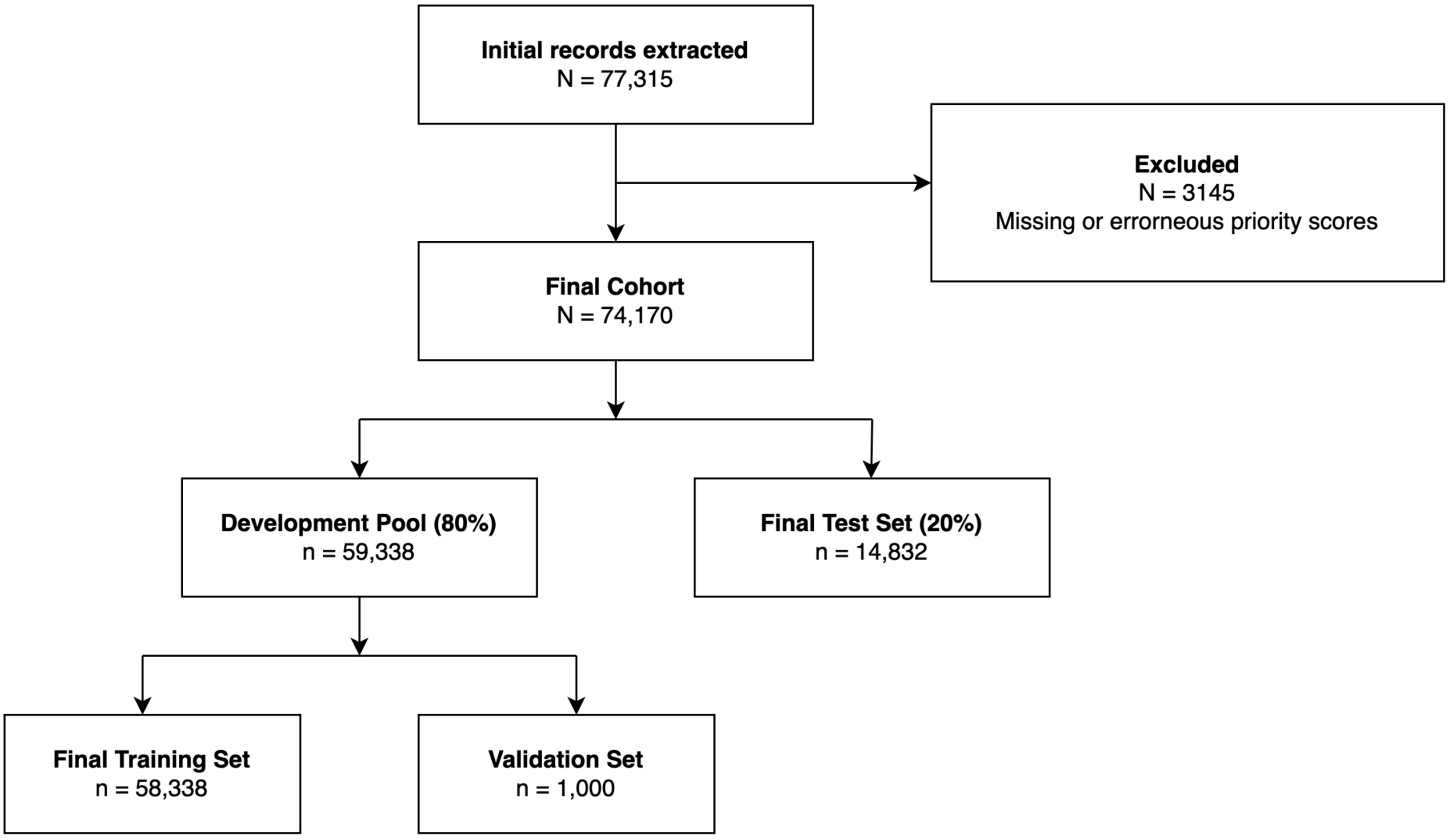
Flowchart of Patient Selection and Dataset Partitioning.

A total of 77,315 triage records were extracted from the hospital database. After exclusion of 3,145 encounters that did not meet cohort eligibility criteria or contained missing or clearly erroneous key triage fields, the final study cohort comprised 74,170 encounters. Cohort selection and dataset partitioning are summarized in Fig 1. The predefined test split contained 14,832 encounters. Within the common evaluable subset, the median age was 52 months (IQR 21–108), and 56.2% were male. The KTAS distribution was as follows: KTAS 3 (45.03%), KTAS 4 (38.40%), KTAS 2 (9.56%), KTAS 5 (5.32%), and KTAS 1 (1.69%). Baseline characteristics and ‌‌class distribution of the evaluable cohort are summarized in Table 1.

**Table 1:** Baseline characteristics and KTAS distribution of the evaluated cohort.

Characteristic	Value (N = 14,832)
Age (months), median (IQR)	52 (21–108)
**Sex, n (%)**	
Male	8,332 (56.2%)
Female	6,500 (43.8%)
**Age group, n (%)**	
Infant (<1 y)	2,019 (13.6%)
Toddler/preschool (1–5 y)	6,101 (41.1%)
School age (6–12 y)	4,370 (29.5%)
Adolescent (13–17 y)	2,221 (15.0%)
Adult/other (≥18 y)	121 (0.8%)
**KTAS level, n (%)**	
KTAS 1 (most urgent)	251 (1.7%)
KTAS 2	1,418 (9.6%)
KTAS 3	6,679 (45.0%)
KTAS 4	5,695 (38.4%)
KTAS 5 (least urgent)	789 (5.3%)

IQR, interquartile range; KTAS, Korean Triage and Acuity Scale.

### Main benchmark results

On the common evaluable test subset, the fine-tuned model outperformed both the majority-class baseline and the pre-fine-tuning model on the global performance metrics shown in Table 2. The majority-class baseline, which assigned KTAS 3 to all encounters, achieved an accuracy of 45.03%, a macro-F1 of 0.124, and a QWK of 0.000. The pre-fine-tuning Qwen3-8B model achieved an accuracy of 35.01%, a macro-F1 of 0.250, and a QWK of 0.229, with a parse failure rate of 2.3% and a within- ± 1-level agreement of 86.08%.

**Table 2:** Benchmark performance of the majority-class baseline, pre-fine-tuning model, structured-data XGBoost comparator, and fine-tuned model on the common evaluable test subset.

Metric	Majority-class baseline	Pre-fine-tuning model	XGBoost comparator	Fine-tuned model
Accuracy, %	45.03	35.01	69.40 [68.72–70.09]	58.60 [57.78–59.40]
Macro-F1	0.124	0.250	0.618 [0.604–0.632]	0.417 [0.404–0.431]
Quadratic weighted kappa	0.000	0.229	0.651 [0.641–0.663]	0.535 [0.523–0.547]
Within ±1 level, %	92.99	86.08	95.33 [94.98–95.63]	97.13 [96.87–97.39]
Parse failure rate	N/A	2.3%	0%	0.01%

Values are percentages unless otherwise indicated. Bootstrap 95% confidence intervals (500 resamples, percentile method) are shown for the XGBoost comparator and the fine-tuned model primary metrics; the majority-class baseline and pre-fine-tuning model are reported as point estimates. Within ±1 level indicates predictions within one KTAS level of the reference. N/A, not applicable. CI, confidence interval; KTAS, Korean Triage and Acuity Scale; QWK, quadratic weighted kappa.

In the direct comparison on the common evaluable test subset, XGBoost achieved an accuracy of 69.40%, a macro-F1 of 0.618, and a QWK of 0.651, whereas the fine-tuned model achieved an accuracy of 58.60%, a macro-F1 of 0.417, and a QWK of 0.535 (Table 2).

Within- ± 1-level agreement was 97.13% for the fine-tuned model and 95.33% for XGBoost. Parse failure rates were 0% for XGBoost and 0.01% for the fine-tuned model.

### Per-class performance and confusion patterns

At the class level, XGBoost showed higher F1 scores than the fine-tuned model across all five triage levels. In the fine-tuned model, performance was strongest in the middle-acuity categories and weaker at both ends of the scale, particularly at level 5. Recall was higher for XGBoost at levels 1, 2, 4, and 5, whereas the fine-tuned model showed slightly higher recall only at level 3 (Table 3).

**Table 3:** Per-class precision, recall, and F1 for XGBoost and the fine-tuned model on the common evaluable test subset (N = 14,832).

	XGBoost comparator			Fine-tuned model		
KTAS level	Precision	Recall	F1	Precision	Recall	F1
KTAS 1	0.639	0.522	0.575	0.571	0.382	0.458
KTAS 2	0.505	0.622	0.557	0.291	0.281	0.286
KTAS 3	0.759	0.692	0.724	0.587	0.711	0.643
KTAS 4	0.726	0.740	0.733	0.664	0.600	0.630
KTAS 5	0.453	0.567	0.503	0.604	0.037	0.069
**Macro avg**	**0.616**	**0.628**	**0.618**	**0.543**	**0.402**	**0.417**

**Fig 2:**
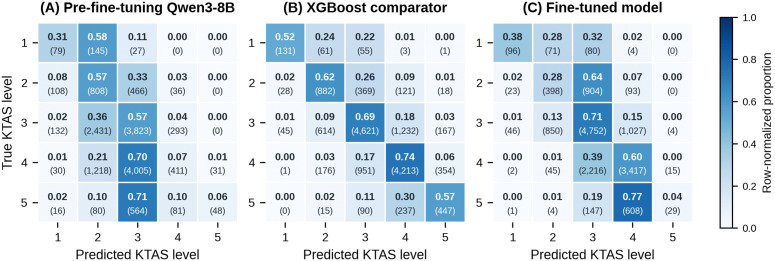
Confusion matrices for pediatric KTAS triage prediction. A) Pre-fine-tuning Qwen3-8B. (B) XGBoost structured-data comparator. (C) Fine-tuned model. Rows represent the reference KTAS levels and columns represent the predicted KTAS levels. Cell values show row-normalized proportions with raw counts in parentheses. All panels use an identical color scale. KTAS, Korean Triage and Acuity Scale.

The confusion matrices showed that, before fine-tuning, predictions were concentrated at levels 2 and 3. After fine-tuning, errors in both XGBoost and the fine-tuned model occurred predominantly between adjacent levels (Fig 2). In the fine-tuned model, true level 1 cases were most often classified as levels 1, 2, or 3, whereas true level 5 cases were most often classified as level 4, consistent with the low recall for level 5. Overall, these class-level results indicate that the fine-tuned model performed best in the middle-acuity range, whereas XGBoost showed more balanced performance across all five levels.

### Safety outcome comparison

On the common evaluable test subset, overall strict under-triage, defined as true levels 1–2 predicted as levels 4–5, was 0.65% (97/14,832) for the fine-tuned model and 0.96% (143/14,832) for XGBoost. Among true level 1 cases, strict under-triage was 1.59% (4/251) in both models. Among true level 2 cases, it was 6.56% (93/1,418) for the fine-tuned model and 9.80% (139/1,418) for XGBoost.

Errors across the emergent/non-emergent boundary also differed between models. True emergent cases, defined as levels 1–3, classified as non-emergent, defined as levels 4–5, occurred in 7.61% (1,128/14,832) of cases with the fine-tuned model and 10.40% (1,542/14,832) of cases with XGBoost. Conversely, true non-emergent cases, defined as levels 4–5, classified as emergent, defined as levels 1–3, occurred in 16.28% (2,415/14,832) of cases with the fine-tuned model and 8.31% (1,233/14,832) of cases with XGBoost.

Overall under-triage was 14.82% for the fine-tuned model and 16.05% for XGBoost, whereas overall over-triage was 26.58% and 14.54%, respectively. Within- ± 1-level agreement was 97.13% for the fine-tuned model and 95.33% for XGBoost, and extreme error rates were 2.87% and 4.67%, respectively (Table 4).

**Table 4:** Safety-relevant error rates for the structured-data XGBoost comparator and the fine-tuned model on the common evaluable test subset (N = 14,832).

Metric	XGBoost comparator	Fine-tuned model
***Strict under-triage***		
Overall	143 (0.96% [0.81-1.12])	97 (0.65% [0.53-0.79])
Among true KTAS 1	4/251 (1.59% [0.17-3.22])	4/251 (1.59% [0.36-3.25])
Among true KTAS 2	139/1,418 (9.80% [8.20-11.39])	93/1,418 (6.56% [5.32-7.85])
***Misclassification across the emergent/non-emergent boundary***		
Emergent-to-non-emergent (KTAS 1–3 → 4–5)	1,542 (10.40% [9.91-10.89])	1,128 (7.61% [7.17-8.01])
Non-emergent-to-emergent (KTAS 4–5 → 1–3)	1,233 (8.31% [7.85-8.77])	2,415 (16.28% [15.73-16.83])
***Overall under-triage and over-triage***		
Over-triage	2,157 (14.54% [14.00-15.15])	3,942 (26.58% [25.92-27.30])
Under-triage	2,381 (16.05% [15.49-16.60])	2,198 (14.82% [14.30-15.40])
***Ordinal agreement***		
Within ±1 level	14,140 (95.33% [94.95-95.66])	14,406 (97.13% [96.86-97.38])
Extreme error (≥2 levels)	692 (4.67% [4.31-4.97])	426 (2.87% [2.59-3.13])

Values are shown as n (% [95% CI]) unless otherwise indicated. Confidence intervals were estimated using 500-resample percentile bootstrap. Strict under-triage was defined as true KTAS 1–2 cases predicted as KTAS 4–5. Misclassification across the emergent/non-emergent boundary includes true KTAS 1–3 cases predicted as KTAS 4–5 and true KTAS 4–5 cases predicted as KTAS 1–3. KTAS, Korean Triage and Acuity Scale.

### Case-level review of strict under-triaged true KTAS 1 encounters

Among the four true KTAS 1 encounters that were strictly under-triaged by the fine-tuned model, all were assigned KTAS 4 and none were assigned KTAS 5. Three cases involved toddlers aged 1–3 years presenting with vomiting, poor oral intake, or foot laceration, with stable recorded body temperature, oxygen saturation, and blood pressure at triage. The remaining case involved an 18-year-old patient with a history of mood disorder whose chief complaint included the ambiguous abbreviation “DI,” which was interpreted as diarrhea rather than drug ingestion. Across these four cases, strict under-triage occurred in the setting of apparently stable recorded triage vital signs, and one case additionally involved an ambiguous abbreviated chief complaint.

### Exploratory subgroup analyses by age and presenting symptom

Across age groups, XGBoost showed higher accuracy and QWK than the fine-tuned model, although both models performed best in younger children. Within the fine-tuned model, strict under-triage was highest in infants (1.14%) and adolescents (0.86%) and lower in the toddler/preschool and school-age groups (0.51%–0.53%) (Table 5).

**Table 5:** Exploratory subgroup performance by age group on the common evaluable test subset (N = 14,832).

Age group (n)	XGBoost accuracy, %	XGBoost QWK	Fine-tuned model accuracy, %	Fine-tuned model QWK	Fine-tuned model strict under-triage, n (%)
Infant, < 1 y (2,019)	72.7 [70.7–74.6]	0.678 [0.646–0.711]	57.1 [55.0–59.6]	0.580 [0.548–0.610]	23 (1.14%)
Toddler/preschool, 1–5 y (6,101)	73.6 [72.5–74.6]	0.679 [0.661–0.697]	62.0 [60.9–63.2]	0.568 [0.551–0.585]	31 (0.51%)
School age, 6–12 y (4,370)	65.1 [63.5–66.6]	0.595 [0.568–0.622]	54.8 [53.5–56.1]	0.439 [0.415–0.467]	23 (0.53%)
Adolescent, 13–17 y (2,221)	63.7 [61.6–65.7]	0.536 [0.499–0.571]	58.7 [56.6–60.9]	0.419 [0.383–0.450]	19 (0.86%)
Adult/other, ≥ 18 y (121)	64.5 [55.4–72.7]	0.511 [0.353–0.660]	50.4 [41.7–57.9]	0.384 [0.216–0.527]	1 (0.83%)

Values are accuracy and quadratic weighted kappa (QWK) with percentile-based 95% bootstrap confidence intervals (500 resamples) for XGBoost and the fine-tuned model. Fine-tuned model strict under-triage denotes true KTAS 1–2 cases predicted as KTAS 4–5 within each age group. Subgroup analyses are exploratory and were not intended for formal between-group statistical comparison. CI, confidence interval; KTAS, Korean Triage and Acuity Scale; QWK, quadratic weighted kappa.

Across presenting-symptom categories, XGBoost showed higher accuracy than the fine-tuned model in most groups, whereas trauma/injury was the only major category in which the fine-tuned model showed comparable accuracy. In the fine-tuned model, fever presentations showed no strict under-triage but high over-triage, whereas neurological presentations showed the highest over-triage rate (S3 Table in S1 File). Because several symptom categories contained sparse representation of rare KTAS levels, these results were interpreted as exploratory descriptive findings.

### Post-hoc training-duration and class-rebalancing analysis

**Fig 3:**
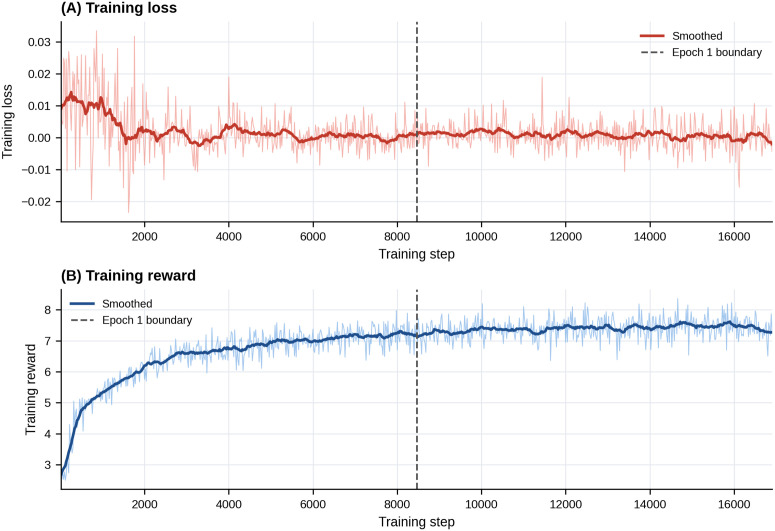
Training dynamics of the post-hoc 2-epoch class-rebalanced GRPO run. (A) Training loss across optimization steps. (B) Training reward across optimization steps. The dashed vertical line indicates the approximate one-epoch boundary. GRPO, Group Relative Policy Optimization; KTAS, Korean Triage and Acuity Scale.

To further examine the effect of longer training with class rebalancing, we conducted a secondary GRPO run with class rebalancing and extended training to two epochs (Fig 3).

The 2-epoch class-rebalanced run showed lower global discrimination than the primary 1-epoch model trained on the natural class distribution (S4 Table in S1 File). Accuracy decreased from 58.60% to 36.07%, macro-F1 from 0.417 to 0.307, and QWK from 0.535 to 0.326. This decline was most evident in the lower-acuity classes, with KTAS 4 recall of 27.6% and KTAS 5 recall of 11.3% in the secondary run. Because this post-hoc analysis changed both training duration and class distribution, the 1-epoch model trained on the natural class distribution was retained as the primary benchmark model.

## Discussion

### Principal findings

In this study, we fine-tuned an open-weight 8-billion-parameter small language model with safety-oriented optimization for pediatric emergency triage and benchmarked it against a structured-data XGBoost comparator. XGBoost showed higher overall discrimination, whereas the fine-tuned model exhibited a different error profile, with greater within- ± 1-level agreement, fewer extreme errors, and lower strict under-triage, offset by higher over-triage. These findings suggest that the behavior of a fine-tuned model in pediatric emergency triage depends not only on its overall classification performance but also on the clinical priorities embedded in model optimization.

### Comparison with baselines

Compared with both the majority-class baseline and the pre-fine-tuning model, the fine-tuned model showed clear improvement in overall accuracy, macro-F1, and QWK. Relative to the majority-class baseline, these gains indicate that the model did not simply collapse to the dominant KTAS 3 class in this imbalanced five-level task. Relative to the pre-fine-tuning model, the improvement suggests that task-specific fine-tuning materially improved alignment with the ordinal structure of KTAS. In this setting, class-sensitive metrics such as macro-F1 and QWK are particularly informative because overall accuracy alone may understate meaningful differences in imbalanced multiclass performance [36]. The high within- ± 1-level agreement also suggests that many errors occurred between adjacent KTAS categories rather than as grossly discordant shifts, which is plausible in KTAS, where intermediate category boundaries are often clinically ambiguous and interrater agreement is imperfect even among experienced raters [30,37].

The structured-data XGBoost comparator nevertheless achieved higher overall discrimination than the fine-tuned model, suggesting that structured triage variables retained substantial predictive signal for this task [13]. Even so, more deployable language-model approaches may still offer practical advantages, including local governance of sensitive data and adaptation to institution-specific documentation patterns and patient populations [19,28,38]. Because model selection in clinical workflows must balance predictive performance against operational constraints [26,27], the most plausible near-term role of the present fine-tuned model is adjunctive decision support rather than replacement of stronger structured-data approaches or clinician judgment [39].

### Safety-oriented error profile

Under-triage and over-triage are competing error modes in any triage system, and the balance between them depends on institutional capacity, crowding, and tolerance for missed high-acuity cases [1,40]. In this study, the fine-tuned model showed lower rates of selected hazardous under-triage outcomes than the structured-data comparator, but this occurred alongside higher over-triage. The higher over-triage rate also has operational implications. In a high-volume pediatric emergency department, a shift toward higher-acuity predictions could redirect a substantial number of lower-acuity patients toward higher-acuity care areas, potentially increasing crowding, staff workload, and resource strain. This pattern is consistent with the safety-oriented reward design used during training and illustrates how model behavior can shift according to the relative weighting of different error types.

This pattern should not be interpreted as the fine-tuned model being uniformly safer. Although strict under-triage was lower in selected high-acuity categories, clinically important misses remained, and overall over-triage was substantially higher. The model also showed limited performance in the highest-acuity group, indicating that aggregate safety metrics alone may not fully capture concerning errors in the sickest children. This limitation is clinically important because recall for true level 1 encounters was only 38.2% in the fine-tuned model. Published validation studies of established triage systems generally report substantially higher sensitivity for high-urgency pediatric presentations, often in the range of 70–80% or higher, although estimates vary by system, population, and reference ‌‌standard [41,42]. Therefore, the present model is not suitable for autonomous identification of the highest-acuity pediatric cases and should only be considered, if at all, as an adjunctive review tool with mandatory human oversight.

The case review further suggests limitations of text-centric triage modeling. High-acuity presentations may depend on visual appearance, subtle work of breathing, evolving clinical trajectory, or clinician gestalt, and abbreviation-heavy clinical notes may further obscure severity signals. More generally, narrative-based representations may fail to capture clinical context that is important for reliable pediatric triage decisions [9]. Accordingly, even a safety-oriented fine-tuned model should be regarded as requiring explicit human oversight and conservative deployment safeguards.

### Comparison with related work

Recent AI-based triage research has expanded beyond rule-based or structured-only systems to include both language-model approaches and hybrid architectures. Recent multi-architecture studies in emergency department triage reported substantially higher performance than that observed in the present study [11,12]. Other published language-model triage studies have also reported encouraging results, but the literature remains heterogeneous in study design and task formulation. Taken together, prior studies and recent reviews indicate that numerical comparisons across reports are difficult to interpret unless population, label source, task formulation, and deployment setting are closely aligned [9,16,43]. In particular, because the present model was trained and evaluated exclusively on KTAS-labeled triage narratives from a Korean pediatric emergency department, its performance should not be assumed to generalize directly to other triage scales, documentation conventions, or linguistic settings without separate evaluation.

Several methodological differences may partly explain the performance gap between these reports and the present study. The prior multi-architecture study evaluated adult emergency department triage using the FRENCH scale and hybrid pipelines that combined language representations with structured-data classifiers, whereas the present study evaluated a single fine-tuned open-weight model in pediatric KTAS triage using routine triage narratives and structured fields [11,12]. In addition, differences in analytic cohort selection, high-acuity class representation, and reference label construction, including expert-consensus labels versus routine nurse-assigned labels, may have contributed to the higher reported performance in prior work.

Against that background, the contribution of the present study is not direct numerical competition with prior reports, but a pediatric benchmark of a fine-tuned open-weight model using routine triage narratives, matched comparison with a structured-data XGBoost comparator, and explicit evaluation of safety-related error patterns. These findings also support further evaluation of hybrid approaches in future work.

### Reproducibility under deterministic inference

The perfect agreement across repeated inference runs in this study reflects the deterministic decoding configuration used at evaluation rather than robustness to stochastic perturbation. Because temperature was set to 0, identical inputs yielded identical outputs. The observed 100% exact agreement should therefore be interpreted as deterministic reproducibility, not as evidence that the model is free from systematic error. Even so, reproducible outputs may still be relevant for clinical decision support because they simplify audit trails, case review, and quality assurance in patient-care AI systems [16,17].

### Label quality and inter-rater agreement

An additional consideration in interpreting these results is the quality of the reference label. In this study, the reference label was the nurse-assigned KTAS level recorded during routine triage rather than an expert-adjudicated label. Although KTAS is standardized and widely used, prior studies have shown that inter-rater agreement is imperfect, particularly around intermediate triage boundaries such as those between KTAS 2 and 3 and between KTAS 3 and 4 [30,37]. Accordingly, model performance may have been constrained in part by label noise, and some adjacent-level disagreements may reflect borderline cases rather than clear model error. This does not lessen the importance of major high-acuity misses, but it does caution against over-interpreting moderate overall discrimination and class-level error rates. Because institution-specific inter-rater reliability data were not available for the present dataset, future studies should incorporate prospective inter-rater assessment and expert adjudication of selected borderline encounters to better distinguish label disagreement from true model error.

### Post-hoc ablation and training dynamics

To examine whether longer training with class rebalancing materially changed model behavior, we conducted a secondary post-hoc GRPO run using rebalanced training data and extending training to two epochs. Compared with the primary 1-epoch model trained on the natural class distribution, the secondary run showed lower global discrimination, with decreases in accuracy, macro-F1, and quadratic weighted kappa. The decline was most evident in the lower-acuity classes.

A plausible explanation is that rebalanced training, applied together with longer optimization and a reward design that penalized under-triage more strongly than over-triage, shifted the model toward higher-acuity predictions. This may have improved sensitivity to rarer high-acuity patterns, but at the cost of poorer discrimination among the more prevalent lower-acuity classes, consistent with the marked decline in recall at levels 4 and 5 and the increase in overall over-triage. Because training duration and class distribution were changed together in this post-hoc run, the relative contribution of each factor cannot be determined. The 1-epoch model trained on the natural class distribution was therefore retained as the primary benchmark model.

### Limitations and future directions

Several limitations should temper interpretation of the present findings. First, this was a retrospective single-center study, and external validation is necessary before generalization to institutions with different patient populations, staffing arrangements, documentation practices, and KTAS use patterns. In addition, the study period spanned January 2020 to April 2025 and therefore included the COVID-19 pandemic and post-pandemic periods, during which pediatric emergency department volumes, respiratory presentations, trauma patterns, and triage behavior may have changed. Because the dataset was randomly partitioned rather than chronologically separated, temporal mixing may have reduced apparent distributional shift between training and test data and may overestimate generalizability to future periods. External and temporally separated validation will be needed to assess robustness under changing case-mix conditions. Second, the reference standard was routine nurse-assigned KTAS rather than expert-adjudicated consensus, so label noise and inter-rater variability may have constrained the attainable agreement ceiling and contributed to some adjacent-level disagreements. Third, the development-stage validation subset was relatively small for a five-class, highly imbalanced task, leaving limited high-acuity signal for tuning reward weights and training duration. Fourth, the model was restricted to triage-time documentation and a limited set of structured fields; clinically important cues such as visual appearance, work of breathing, evolving trajectory, and clinician gestalt were unavailable, and abbreviation-heavy clinical notes may have further obscured severity signals [9]. Fifth, subgroup analyses suggested possible heterogeneity by age group and presenting symptom, but these findings were exploratory and some subgroup estimates were based on comparatively small samples; they should therefore be interpreted as hypothesis-generating rather than as definitive between-group differences [41,42]. Finally, the comparison between the fine-tuned model and XGBoost should be interpreted in light of differences in input space. The fine-tuned model used reconstructed case-level text combining structured variables and nurse-authored narratives, whereas XGBoost used structured variables only. Therefore, the present benchmark ensured evaluation on the same held-out test subset but did not quantify the incremental contribution of the narrative component within the language-model pipeline. Future ablation studies comparing structured-only, narrative-only, and combined inputs will be needed to determine whether the performance gap reflects model architecture, fine-tuning strategy, input representation, or limited additional signal in the narrative text.

Future work should prioritize external and prospective validation, expert review of selected high-acuity or borderline cases, and comparison with hybrid models that combine narrative and structured triage signals. Additional work is also needed to refine reward calibration and practical safeguards for ambiguous or abbreviation-heavy clinical notes. Until such evidence is available, the present model should be regarded as a decision-support aid for human triage review rather than a substitute for clinician judgment.

## Conclusions

In this real-world pediatric emergency department benchmark, the structured-data XGBoost comparator showed higher overall performance than the fine-tuned model. The fine-tuned model nevertheless showed a distinct error profile, with fewer extreme errors and lower strict under-triage in selected high-acuity groups, at the cost of higher over-triage. These findings suggest that the present fine-tuned model may be more appropriately considered as a decision-support aid for human triage review rather than as an autonomous triage system. External and prospective validation will be necessary before clinical implementation.

## Supporting information

S1 File Supplementary tables. (DOCX)
