# Supplementary material for: Performance and safety of a fine-tuned small language model for pediatric emergency triage: A benchmark study
Source: PLoS One. 2026 Jun 4;21(6):e0350770. doi: 10.1371/journal.pone.0350770 (PMC13235901; doi:10.1371/journal.pone.0350770)
Supplement: S1 File — (DOCX) [file pone.0350770.s001.docx]

**S1 Table. GRPO Fine-tuning Hyperparameter Configuration**

Configuration used for primary 1-epoch and post-hoc 2-epoch GRPO runs. Parameters shared by both runs appear once; run-specific parameters are noted.

| **Section** | **Parameter** | **Value** |
| --- | --- | --- |
| **Base model** | Model name | Qwen3-8B-Base |
|  | Floating-point precision | bfloat16 (LoRA 16-bit training) |
| **LoRA adapter** | Rank (r) | 32 |
|  | Alpha (α) | 64 (α = 2r) |
|  | Dropout | 0.0 |
|  | Target modules | q_proj, k_proj, v_proj, o_proj, gate_proj, up_proj, down_proj |
|  | Trainable parameters | ≈ 83 million |
| **GRPO optimizer** | Optimizer | AdamW (8-bit) |
|  | Learning rate | 5 × 10⁻⁶ |
|  | Weight decay | 0.01 |
|  | Warmup ratio | 0.10 |
|  | LR scheduler | Linear decay |
|  | Per-device batch size | 4 |
|  | Gradient accumulation steps | 4 (effective batch size = 16) |
| **GRPO sampling (during training)** | Completions per prompt (num_generations) | 4 |
|  | Sampling temperature | 1.0 |
|  | Sampling min_p | 0.1 |
| **Primary 1-epoch model** | Training data | Natural class distribution (N = 58,336) |
|  | Training epochs | 1 |
|  | Total training steps  Estimated training time | 13,381  117 hours |
| **Post-hoc 2-epoch model (exploratory)** | Training data | KTAS 1 oversampled ×10; KTAS 2 oversampled ×4 |
|  | Training epochs | 2 |
|  | Total training steps  Estimated training time | 16,906  148 hours |
|  |  |  |
| **System Prompt** | You are a clinical reasoning model specializing in emergency medicine and well-versed in the Korean Triage and Acuity Scale (KTAS) classification system. Your task is to logically determine the appropriate KTAS level based on the clinical information provided about the patient and to present your conclusion. Analyze the case through logical reasoning and determine the correct KTAS level. Think about the problem and provide your working out. Place it between <REASONING> and </REASONING>. Then, provide your solution between <SOLUTION> and </SOLUTION>. The solution must be a single KTAS level number (1–5) only, without any additional words or punctuation.  Below is a general description of KTAS. Do not restate this information during your reasoning process. KTAS is a Korean triage system used to classify the severity and urgency of patients in the emergency department. It consists of 5 levels. Level 1 (Resuscitation): Immediate life- or limb-threatening condition requiring aggressive intervention. Level 2 (Emergent): Potential threat to life or limb requiring prompt intervention under physician guidance. Level 3 (Urgent): Potentially serious condition requiring emergency treatment. Level 4 (Less urgent): Condition requiring treatment or re-evaluation within 1–2 hours. Level 5 (Non-urgent): Acute but non-urgent condition, or a chronic issue with no recent change. | |

GRPO, Group Relative Policy Optimization; KTAS, Korean Triage and Acuity Scale; LoRA, Low-Rank Adaptation; LR, learning rate.

**S2 Table. Reward Function Mapping and Coefficients for GRPO Training**

Rewards and penalties applied to each candidate completion during GRPO training. Component scores are summed to yield the net reward per output.

| **Category** | **Condition** | **Score** | **Clinical/structural rationale** |
| --- | --- | --- | --- |
| **Correctness** | Exact match with reference KTAS level | **+5.0** | Primary optimization target |
| **Safety shaping (under-triage)** | True KTAS 1–2 predicted with \|diff\| = 1 | **−2.0** | Near-miss high-acuity penalty |
|  | True KTAS 1–2 predicted with \|diff\| ≥ 2 | **−4.0** | Strict under-triage; major safety failure |
|  | True KTAS 3–4 predicted with \|diff\| = 1 | **−1.5** | Moderate mid-acuity under-triage penalty |
|  | True KTAS 3–4 predicted with \|diff\| ≥ 2 | **−3.0** | Strong mid-acuity under-estimation penalty |
| **Resource shaping (over-triage)** | Any level predicted with diff = −1 | **−0.5** | Mild over-triage deterrent |
|  | Any level predicted with diff ≤ −2 | **−2.0** | Stronger over-triage deterrent |
| **Bias mitigation** | Predicting KTAS 3 when ground truth ≠ KTAS 3 | **−0.5** | Counteracts majority-class collapse |

GRPO, Group Relative Policy Optimization; KTAS, Korean Triage and Acuity Scale. Positive scores are rewards; negative scores are penalties.

**S3 Table. Exploratory subgroup performance by presenting symptom category on the common evaluable test subset (N = 14,832)**

Symptom categories assigned by hierarchical keyword matching (priority: Neurological > Respiratory > Gastrointestinal > Trauma/injury > Dermatology > ENT/Eye > Metabolic/Endocrine > Fever > Other). Accuracy values include percentile-based 95% bootstrap CIs (500 resamples). Strict Under-triage: true KTAS 1–2 predicted as KTAS 4–5 (proportion of total subgroup).

| **Symptom category** | **n** | **XGBoost accuracy, %** | **XGBoost strict under-triage** | **Fine-tuned model accuracy, %** | **Fine-tuned model strict under-triage** | **Fine-tuned model over-triage** |
| --- | --- | --- | --- | --- | --- | --- |
| Fever | 3,555 | 74.5 [73.2–75.9] | 13 (0.37%) | 52.7 [51.1–54.4] | 0 (0.00%) | 1,255 (35.3%) |
| Gastrointestinal | 3,114 | 66.3 [64.7–68.0] | 23 (0.74%) | 61.1 [59.5–62.8] | 25 (0.80%) | 681 (21.9%) |
| Other | 2,805 | 66.4 [64.6–68.1] | 11 (0.39%) | 59.9 [58.1–61.5] | 3 (0.11%) | 692 (24.7%) |
| Trauma/injury | 2,215 | 76.7 [75.1–78.3] | 57 (2.57%) | 73.1 [71.4–75.1] | 29 (1.31%) | 343 (15.5%) |
| Respiratory | 940 | 61.5 [58.2–64.5] | 3 (0.32%) | 57.2 [54.3–60.5] | 3 (0.32%) | 216 (23.0%) |
| Neurological | 908 | 75.0 [72.2–77.8] | 3 (0.33%) | 44.1 [40.9–47.4] | 6 (0.66%) | 346 (38.1%) |
| Dermatology | 792 | 53.2 [49.7–56.7] | 21 (2.65%) | 46.5 [42.9–50.0] | 19 (2.40%) | 302 (38.1%) |
| ENT/Eye | 478 | 66.7 [62.3–71.2] | 12 (2.51%) | 61.3 [56.9–65.7] | 11 (2.30%) | 105 (22.0%) |
| Metabolic/Endocrine | 25 | 72.0 [56.0–88.0] | 0 (0.00%) | 60.0 [40.0–76.0] | 1 (4.00%) | 2 (8.0%) |

Symptom categories were assigned by hierarchical keyword matching. Values are accuracy with percentile-based 95% bootstrap confidence intervals (500 resamples) for XGBoost and the fine-tuned model. Over-triage denotes prediction of a higher urgency level than the reference label. Subgroup analyses are exploratory. ENT, ear/nose/throat; KTAS, Korean Triage and Acuity Scale.

**S4 Table. Performance across model training stages: pre-fine-tuning, primary 1-epoch, and post-hoc 2-epoch run**

| **Metric** | **Pre-fine-tuning model** | **Primary fine-tuned model** | **Post-hoc 2-epoch rebalanced run** |
| --- | --- | --- | --- |
| ***Global discrimination*** |  |  |  |
| Accuracy, % | 35.01 | 58.60 [57.78–59.40] | 36.07 [35.31–36.82] |
| Macro-F1 | 0.250 | 0.417 [0.404–0.431] | 0.307 [0.297–0.319] |
| Quadratic weighted kappa | 0.229 | 0.535 [0.523–0.547] | 0.326 [0.315–0.339] |
| ***Ordinal agreement*** |  |  |  |
| Within ±1 level, % | 86.08 | 97.13 | 85.27 [84.71–85.84] |
| Extreme error (≥2 levels), % | 13.92 | 2.87 | 14.73 [14.20–15.28] |
| ***Overall under-triage and over-triage*** |  |  |  |
| Over-triage, % | 58.55 | 26.58 | 54.75 |
| Under-triage, % | 6.45 | 14.82 | 9.18 |
| ***Class-level recall, %*** |  |  |  |
| KTAS 1 | 31.6 | 38.2 | 54.3 |
| KTAS 2 | 57.0 | 28.1 | 63.5 |
| KTAS 3 | 57.2 | 71.1 | 39.7 |
| KTAS 4 | 7.2 | 60.0 | 27.6 |
| KTAS 5 | 6.1 | 3.7 | 11.3 |

Bootstrap 95% confidence intervals (500 resamples, percentile method) are shown for the primary fine-tuned model and the post-hoc 2-epoch run; the pre-fine-tuning model is reported as a point estimate. The post-hoc run used class-rebalanced training data and deterministic decoding at evaluation. CI, confidence interval; GRPO, Group Relative Policy Optimization; KTAS, Korean Triage and Acuity Scale.
